# Prevalence of bacterial vaginosis, sexually transmitted infections and their association with HPV infections in asymptomatic women attending antenatal care in Ethiopia

**DOI:** 10.3332/ecancer.2024.1783

**Published:** 2024-09-30

**Authors:** Johanna M. A. Klein, Isabel Runge, Ann-Katrin Pannen, Tariku Wakuma, Semaw Ferede Abera, Adamu Adissie, Susanne Unverzagt, Markus Schmitt, Tim Waterboer, Daniela Höfler, Christoph Thomssen, Eva Johanna Kantelhardt

**Affiliations:** 1 Department of Gynaecology, Martin Luther University Halle-Wittenberg, Halle (Saale) 06097, Germany; 2 Global Health Working Group, Martin Luther University Halle-Wittenberg, Halle (Saale) 06097, Germany; 3 Aira Hospital, Aira, Ethiopia; 4 Department of Radiation Oncology, Faculty of Medicine, Martin Luther University Halle-Wittenberg, Halle (Saale) 06097, Germany; 5 School of Public Health, College of Health Sciences, Mekelle University, Mekelle, Ethiopia; 6 Kilte Awlaelo - Health and Demographic Surveillance Site, College of Health Sciences, Mekelle University, Mekelle, Ethiopia; 7 School of Public Health, College of Health Sciences, Addis Ababa University, Addis Ababa 1000, Ethiopia; 8 Institute of General Practice and Family Medicine, Center of Health Sciences, Martin Luther University Halle-Wittenberg, Halle (Saale) 06097, Germany; 9 Infections and Cancer Epidemiology, German Cancer Research Center (DKFZ), Heidelberg 69120, Germany

**Keywords:** sexually transmitted diseases, bacterial vaginosis, Ethiopia, pregnancy, vaginal lavage, human papillomavirus

## Abstract

Sexually transmitted infections (STIs) and human papillomavirus (HPV) infections are common among women of reproductive age and can lead to infertility, adverse pregnancy outcomes, neonatal infections and cervical cancer. In countries with limited medical coverage, untreated infections contribute to high morbidity. This study aimed to expand the current knowledge on the prevalence of bacterial vaginosis (BV) and STIs in pregnant Ethiopian women and assess the association of these conditions with HPV infections. Socio-demographic data and vaginal lavage samples were collected from 779 asymptomatic women aged 18 to 45 years (median age, 25.9 years) attending antenatal care in seven centres across Ethiopia. Multiplex polymerase chain reaction was used to test for BV, *Chlamydia trachomatis*, *Trichomonas vaginalis*, *Neisseria gonorrhoeae*, herpes simplex virus types 1 and 2 (HSV-1/2), *Mycoplasma*, *Ureaplasma*, *Candida species* and HPV. Overall, 26.8% (95% confidence interval (CI): 23.7–29.9) of women tested positive for BV or one of the following STIs: *C. trachomatis*, *T. vaginalis*, *N. gonorrhoeae*, *Mycoplasma genitalium*, HSV-1/2 or *Ureaplasma urealyticum*. Additionally, 22.1% tested positive for at least one high-risk HPV type. *Chlamydia trachomatis* and HSV-2 were significantly more common among women who were positive for HPV and high-risk HPV. This study reveals a high prevalence of asymptomatic pregnant women who are positive for BV, STIs or HPV, putting them at risk of adverse pregnancy outcomes, secondary infertility or cervical cancer in a country with limited medical coverage. Screening and treating these women could be crucial in reducing morbidity.

## Background

Bacterial vaginosis (BV) and sexually transmitted infections (STIs) are common among women of reproductive age. The association between BV and adverse pregnancy outcomes is a subject of ongoing debate [1]. Infections with *Chlamydia trachomatis*, *Neisseria gonorrhoeae*, *Treponema pallidum*, herpes simplex virus type 2 (HSV-2) and *Ureaplasma urealyticum* are strongly linked to adverse pregnancy outcomes, including miscarriages, premature rupture of membranes, preterm labour and delivery, neonatal infections, low birth weight, puerperal endomyometritis and secondary infertility [2–8]. Delayed diagnosis and treatment, especially in countries with limited medical resources, can lead to serious complications.

BV is characterised by a reduction in lactobacilli and an overgrowth of anaerobic bacteria such as *Gardnerella vaginalis*, *Atopobium vaginae* and *Mycoplasma hominis*, and it is a known risk factor for preterm labour [1]. While these bacteria may not be highly pathogenic on their own, they may facilitate infection by more pathogenic bacteria because the protective role of the physiological lactobacilli vaginal flora is compromised in BV. Consequently, it has been suggested that BV may also facilitate infections with high-risk human papillomavirus (HPV) [9]. High-risk HPV is a necessary, but not sufficient, cause of cervical cancer; however, most infections are transient and cause no or only mild cervical changes [10]. Associations between infections with *C. trachomatis* or HSV-2 and the development of HPV or cervical cancer suggest that these infections may act as cofactors in the development of cervical cancer and precancerous lesions [11–13].

Screening and treating pregnant women is challenging but crucial. Many women are asymptomatic and, therefore, remain untreated, resulting in serious medical complications for both mother and child. Early detection and treatment of BV and STIs may prevent preterm labour and vertical transmission of infections, while in women with high-risk HPV, it can reduce morbidity and mortality due to cervical cancer [12, 14].

A feasible screening method for BV and STIs in low-resource countries is STI profiling (STIP). STIP is a multiplex polymerase chain reaction (PCR) technique that can detect multiple infectious agents simultaneously, requiring only a single vaginal or cervical swab or a lavage sample for testing [15].

Limited data are available on BV, STIs and HPV infection in Ethiopia. The aim of this study was to expand the current knowledge on the prevalence of BV and STIs among asymptomatic young pregnant women in Ethiopia and to evaluate the association of these conditions with HPV infections. Such information will help estimate the number of women at risk for adverse pregnancy outcomes.

## Methods

### Study population

In 2013 and 2014, a cross-sectional study was conducted to test asymptomatic pregnant women for STIs and HPV across seven centres in Ethiopia [16]. To ensure comprehensive data collection, samples were obtained from antenatal care and family planning units at university hospitals, state and private hospitals, and regional health centres throughout the country.

Women aged 18 to 45 years were eligible to participate if they had no signs of placenta praevia, severe pre-eclampsia or other pregnancy-related complications. Women visiting the facility because of vaginal bleeding or discharge were also excluded, although some women showed signs of discharge upon examination (Supplementary Table 1). Of the 1,239 women attending antenatal and family planning units on the enrolment days, 1,041 agreed to participate and completed all study procedures. Due to missing samples and invalid analyses, 262 samples were excluded from the final analysis, leaving 779 valid samples from antenatal care (*n* = 747) and family planning (*n* = 32). Women from seven geographical areas were included in the study: Addis Ababa (*n* = 209), Mekelle (*n* = 74), Bahir Dar (*n* = 215), Harar (*n* = 29), Ginir (*n* = 38), Wukro (*n* = 65) and Aira (*n* = 149).

### Patient involvement

The patients were involved in the design and conduct of this research. During the feasibility stage, the research question, choice of outcome measures and recruitment methods were informed by discussions with the patients through a rapid ethnographic assessment session conducted by the research team.

### Sample size

To detect a minimum 10% difference between the proportion of STI-positive women in rural areas (assumed 14% prevalence and 20% of participants) and urban areas (assumed 24% prevalence and 80% of participants), 845 women were required for analysis (845 total = 169 rural + 676 urban), with an alpha of 5% and a power of 80%, using the chi-squared test. The ratio of rural to urban participants was derived from a preliminary inquiry at the sites.

### Questionnaire

To obtain socio-demographic and reproductive data, local nurses administered a questionnaire that had been previously used in other studies [17]. The questionnaire was slightly modified to suit the study population. It was translated into Amharic, Oromo and Tigrinya and validated by retranslation conducted by native speakers to ensure accuracy and avoid any loss or misinterpretation of information.

### Sample collection

Samples were collected during routine antenatal care and family planning visits. Local nurses provided a brief introduction to STIs, their transmission and their symptoms. The study procedures were explained, informed consent was obtained and codes were assigned to ensure confidentiality.

Vaginal samples were collected using a syringe-like self-sampling device (Delphi Bioscience, Scherpenzeel, Netherlands). The device was inserted into the vagina, releasing 3 mL of buffered saline by plunging the handle. The fluid was then retracted back into the device by releasing the handle. The lavage fluid was transferred into transportation tubes containing 3 mL of buffered methanol solution, where it was preserved for up to 3 months at room temperature (20°C–30°C). Medical staff assisted with the sample collection. The samples were analysed at the German Cancer Research Centre (DKFZ) in Heidelberg, Germany.

### HPV DNA testing

For the detection of HPV DNA in the vaginal lavage samples, a multiplex papillomavirus genotyping assay was performed as previously described [18]. This method analyses 51 HPV types, of which 14 are considered high-risk: HPV 16, 18, 31, 33, 35, 39, 45, 51, 52, 56, 58, 59, 66 and 68a/b [19]. Results were expressed as median fluorescence intensity values. Samples were considered positive if they contained β-globin as a human DNA marker and/or HPV DNA.

### STIP

STIP, is a validated multiplex PCR that simultaneously analyses *Atopobium vaginae*, *Candida albicans*, *Candida glabrata*, *Candida krusei*, *Chlamydia trachomatis*, *Gardnerella vaginalis*, HSV-1/2, *Mycoplasma genitalium*, *Mycoplasma hominis, Mycoplasma pneumoniae, Mycoplasma spermatophilum, Neisseria gonorrhoeae, Treponema pallidum, Trichomonas vaginalis, Ureaplasma urealyticum, Ureaplasma parvum and Lactobacillus iners* [15]*.* All other *Lactobacillus* and *Candida* species were detected using universal probes. To confirm the presence of human DNA, the PolA sequence was analysed. Samples positive for STIs and/or PolA were considered valid. Results are reported in median fluorescence intensity values.

A score for BV (BV-score) was calculated as previously described, based on the ratios of *G. vaginalis* and *A. vaginae* to *Lactobacillus* [15]. The presence of *Mycoplasma hominis* was also taken into account.

### Statistical analysis

All descriptive and statistical analyses were conducted using SPSS software version 23 (IBM Corp., Armonk, NY, USA). The prevalences of STIs and HPV, along with the corresponding 95% CIs, were calculated. Fisher’s exact test was used to determine statistical significance, with *p*-values of <0.05 considered statistically significant.

### Ethical clearance

Ethical clearance was obtained from Addis Ababa University and Martin Luther University Halle in February 2011 and April 2013, respectively.

## Results

### Study population

The participants ranged in age from 18 to 45 years, with a mean age of 25.9 years. All major ethnic and religious groups were represented. Table 1 provides an overview of the demographic and reproductive characteristics of the participants.

One fifth of the women had never lived in a city or town and were classified as rural for this study. The remaining women had lived in urban areas or under urban influence and were therefore classified as urban.

More than half of the women (59.7%) identified as housewives, while 15.2% were government employees and 13.2% were merchants. The remaining women were students, employed in other professions or unemployed.

Of the 747 pregnant women, 10.6% were in the first trimester, 35.3% in the second trimester and 54.1% in the third trimester of pregnancy. Most women (94.9%) were married, and 13.6% reported that their husbands had up to three other wives (polygamy). Before becoming pregnant, many women had used hormonal contraceptive methods (70.6%), but condom use was low (1.5%).

One quarter of the women had previously given birth to between one and eight children. Up to four past spontaneous abortions were reported by 18.5% of the women, while 7.8% had undergone up to three induced abortions. No data on the number of sexual partners were obtained to avoid the risk of response bias due to cultural and confidentiality reasons.

### Prevalence of STIs and HPV

One third of the women (33.0%) tested positive for at least one mucosal HPV type, and 22.1% were positive for at least one high-risk HPV type. Table 2 presents the prevalence of STIs, organisms of the vaginal flora and HPV.

Infections with *C. trachomatis*,* T. vaginalis*,* N. gonorrhoeae*,* M. genitalium* and HSV-1/2 were categorised as STIs. The overall prevalence of these STIs in this population was low.

Only a small number of women tested positive for cervical infections with *C. trachomatis* (0.6%) and *N. gonorrhoeae* (0.6%). Infections with *T. vaginalis* (2.3%) and HSV-2 (1.5%) were detected more frequently but remained relatively low. A total of 6.2% of the women were diagnosed with at least one STI. However, only three women had two concurrent STIs: one woman had *C. trachomatis* and *T. vaginalis*, one had *C. trachomatis* and *M. genitalium*, and one had *M. genitalium* and HSV-2. No woman was positive for more than two STIs.

Among the 145 women who tested positive for BV, 126 had a BV-score of ≥2, indicating an unbalanced vaginal flora.

In total, 209 (26.8%) women were positive for BV (score of ≥2), any STI or *U. urealyticum.*

Women from rural backgrounds were more likely to have a BV-score of >2 (36.5% [95% CI: 26.2–46.7]) than women from urban backgrounds (22.0% [95% CI: 18.1–25.9]). No differences were found in the prevalence of STIs between rural and urban areas. Additionally, no significant differences were observed in the BV or STI prevalence based on age, trimester of pregnancy, educational level, age at sexual debut or circumcision status.

### Correlation between STIs and HPV

The observed correlations between STIs and HPV are shown in Table 3.

*Chlamydia trachomatis* and HSV-2 were significantly more common among women who tested positive for HPV and high-risk HPV, while *M. genitalium* was more frequent in women with HPV infections but not in those with high-risk HPV infections.

Although all three bacteria associated with BV (*A. vaginae*,* G. vaginalis* and *M. hominis*) were significantly associated with both HPV and high-risk HPV, having a positive BV-score (BV-score ≥ 2) was not associated with these infections. Candidiasis was also not associated with HPV. Among other bacteria, only *U. parvum* was found to be associated with HPV infection.

## Discussion

### Summary

This is the first multicentre survey to provide data on a broad spectrum of STIs in pregnant Ethiopian women attending antenatal care. This study included only women who considered themselves asymptomatic and were therefore not included in the syndromic approach to STI treatment, as recommended by the Ethiopian national plan. Among these asymptomatic women, 26.8% tested positive for BV, STIs or *U. urealyticum*, and 33.0% tested positive for HPV.

### Background

According to Molla et al. [20], health-seeking behaviour regarding STIs is poor among young Ethiopians because of a lack of knowledge about STIs and the stigma associated with premarital sexual activities. Poor medical coverage and limited knowledge of treatment options among physicians further complicate the situation [21]. Apart from HIV and haemoglobin testing, no routine tests are performed on pregnant women in Ethiopia.

### BV

BV during pregnancy can lead to premature rupture of membranes and preterm labour but is often asymptomatic, remaining undetected and untreated [1]. In this study, 24.3% of asymptomatic women tested positive for BV, which is consistent with results from different regions in Ethiopia showing BV prevalences of 19.4%–29.4% [22–25]. Although the benefits of treatment during pregnancy are debated, there is evidence supporting early treatment [26]. Unexpectedly, rural women were more likely to test positive for BV. The influence of hygienic conditions and nutritional factors should be explored in these settings.

### HPV

High-risk HPV is a necessary but not sufficient cause of cervical cancer [27]. HPV can be sexually transmitted, and although most people will be infected at some point in their lives, the majority of infections are transient and resolve within months [10]. Young and sexually active individuals are particularly at risk. In this study, 33.0% of the population tested positive for any HPV and 22.1% tested positive for high-risk HPV. These findings are consistent with other studies that reported a 33.6% prevalence of HPV in East Africa and Ethiopia [28, 29].

### Chlamydia trachomatis and N. gonorrhoeae

Cervical infections with *C. trachomatis* and *N. gonorrhoeae* during pregnancy may lead to miscarriages, preterm labour, neonatal infections, postpartum upper genital tract infection and secondary infertility [2–5, 30]. Adequate antimicrobial therapy can prevent these outcomes [30]. In this study, only 0.6% of participants tested positive for *C. trachomatis* and *N. gonorrhoeae*. Studies from around the world have shown higher prevalence rates in the female adult population, ranging from 5.0% to 14.7% for *C. trachomatis* and 0.4% to 6.7% for *N. gonorrhoeae*, with prevalences of 7.8% to 9.8% for *C. trachomatis* and 2.4%–4.3% for *N. gonorrhoeae* in Eastern Africa and Ethiopia [5, 31, 32, 33–35]. Our asymptomatic, fertile and married population might be expected to have a lower prevalence of cervical STIs in general. However, the high prevalence of other vaginal STIs and HPV suggests that the low detection of cervical infections may have been due to limitations in the detection method used, rather than reflecting the actual prevalence.

### Other vaginal infections

The pregnant women in this study also demonstrated various types of vaginal infections, such as *T. vaginalis* (2.3%) and *U. urealyticum* (19.5%), which are suspected to cause preterm labour and placental inflammation [14, 36, 8]. However, treatment appears to be beneficial only for *U. urealyticum* infections, not for trichomoniasis [37, 38]. The prevalence of *M. genitalium* infections (1.0%) in this population is consistent with other studies [31].

Primary infections with HSV-2 during pregnancy are known to cause severe neonatal infections and continue to be a concern [39]. In this study, 1.5% of the women had active HSV-2 infections, although it is unclear whether these were primary or recurrent infections. Fortunately, no cases of active syphilis were detected in this population.

*Candida* infections are common among pregnant women and were detected in 38.6% of women in this study. While the impact of *Candida* infections on pregnancy outcomes is debated, the morbidity associated with recurring* Candida* infections is undisputed [40].

### Correlations of BV and STIs with HPV

Studies have suggested a possible correlation between HPV and BV, which was not observed in this study, although *G. vaginalis*, *A. vaginae* and *M. hominis* were significantly associated with both HPV and high-risk HPV [9]. A significant correlation was found between HPV and *C. trachomatis* and HSV-2, as noted in previous studies [11, 41]. While this correlation could be partly explained by the number of sexual partners, the lack of association between HPV and other STIs makes this an unlikely sole explanation.

In this study, *U. parvum* was associated with HPV (odds ratio, 2.12 [1.55–2.89]). This finding has not been reported in the literature, and further studies are needed to determine whether this is a coincidence.

### Strengths and limitations

The main strength of this study lies in its large sample size of pregnant women from seven urban and rural centres across Ethiopia, capturing the country’s diversity. Additionally, a broad spectrum of bacterial, viral and parasitic STIs was detected using a single sample analysed by PCR, an effective detection method. This study provides a comprehensive picture of the prevalence of BV, STIs and HPV in this pregnant population.

However, this study has certain limitations. The main limitation is the lack of information on recent antibiotic use, which may have reduced the prevalence of vaginal infections. Because we described a routine cohort, we assume this represents a normal population, including a proportion of women who have recently used antibiotics, which is common in the Ethiopian context. Another limitation is the disproportionate overrepresentation of urban women due to the location of the included health centres and possible misperception of the term ‘urban’ by participants from villages.

Although the vaginal lavage and STIP methods are validated, the combination has not been previously used in pregnant women. We were unable to compare this method with routine clinical tests for STIs in the Ethiopian setting. It is possible that due to technical issues, the lavage may not have reached the cervix as intended, potentially leading to an underestimation of cervical infections such as *C. trachomatis* and *N. gonorrhoeae*. Further studies should evaluate this combined method. Additionally, no data on pregnancy outcomes in this population could be obtained because of organisational limitations.

## Conclusion

This study has demonstrated that many asymptomatic pregnant women aged 18 to 45 years have BV or curable STIs, putting them at risk for adverse pregnancy outcomes and secondary infertility. A relatively high proportion of women with potentially pathogenic infections was detected. In settings with poor medical coverage, preterm labour or infection of the unborn child can result in high morbidity or even mortality. Screening and treatment for BV and STIs may prevent these adverse outcomes.

As previous studies have shown, knowledge about STIs in Ethiopia is low [20]. Efforts should be made to educate young people about the transmission, symptoms, consequences and prevention of STIs. Adolescents should be educated at a younger age, preferably before sexual debut. Antenatal care presents a valuable opportunity to educate, screen and treat women for STIs and HPV and should be included in antenatal care protocols.

The self-sampling method is well accepted and could be suitable for low-resource countries. However, the multiplex methodology requires a well-equipped laboratory with expensive technology and specialised laboratory technicians.

Finding adequate screening methods and funding remains a challenge, but it is essential to protect pregnant women and their unborn children.

## Conflicts of interest

The authors declare no conflict of interest.

## Ethical approval

Ethical clearance was obtained from Addis Ababa University, Ethiopia, in February 2011 (124/10/IM;032/2011) and April 2013 (050/2013), and from Martin Luther University Halle-Wittenberg, Germany, on 23 August 2010.

## Informed consent

Verbal informed consent for publication was obtained from the patients, as suggested by the ethics committee due to low literacy rates and since a written consent is only provided concerning marriage or business contracts.

## Authors’ contributions

IR, JMAK, AA and EJK designed the research. JMAK, IR, DHoe, AA, SU, CT and EJK analysed the data. IR, JMAK, DHoe and EJK drafted the manuscript. JMAK, IR, AKP, TW, SFA, AA, MS, TW and DH provided the original samples, laboratory results, information on the respective populations and advice on study design, analysis and interpretation of the findings. All authors provided critical interpretation of the results and reviewed the first draft. All authors read and approved the final manuscript.

## Availability of data and materials

Data are available on reasonable request and can be requested by email.

## Figures and Tables

**Table 1.** Socio-demographic and reproductive data of the study population.

	Total population
	*n* = 779^1^
Age, years	25.9 (18–45)
Background	
Rural	152 (19.5)
Urban	626 (80.5)
Ethnic group	
Amhara	276 (35.5)
Oromo	213 (27.4)
Tigre	141 (18.1)
Gurage	100 (12.9)
Other	48 (6.2)
Religion	
Orthodox	488 (62.6)
Protestant	169 (21.7)
Muslim	119 (15.3)
Other	3 (0.4)
Education	
Illiterate	149 (19.3)
Read and write only	56 (7.2)
Formal education	572 (73.4)
Menarche, years	14.7 (7–20)
Age at sexual debut, years	19.1 (5–33)^2^
Marital status	
Single	22 (2.8)
Married	739 (94.9)
Widowed/divorced	18 (2.3)
Parity	
Nulliparous	370 (47.5)
Primiparous	214 (27.5)
Multiparous	195 (25.0)
Female circumcision - Age at circumcision, years	436 (56.5) 7.4 (0–20)
Polygamy	105 (13.6)

Data are presented as median (range) or *n* (%).

1 *n* refers to total number of women in this category. It varies from line to line because of missing answers in the questionnaire. The maximum was eight missing answers in one category (circumcision). All listed percentages take this into account.

2 One woman reported rape at the age of 5 years.

**Table 2.** Prevalence of STIs and HPV.

Organism	*n*	% (95% CI)	Total *n*
HPV			
Any HPV	257	33.0 (29.7–36.3)	779
High-risk HPV^1^	172	22.1 (19.2–25.0)	779
STI			
*C. trachomatis*	5	0.6 (0.1–1.2)	779
*T. vaginalis*	18	2.3 (1.3–3.4)	779
*N. gonorrhoeae*	5	0.6 (0.1–1.2)	779
*M. genitalium*	8	1.0 (0.3–1.7)	779
HSV-2	12	1.5 (0.7–2.4)	779
HSV-1	3	0.4 (0.1–1.2)	779
STI-positive^2^	48	6.2 (4.5–7.9)	779
BV			
*A. vaginae*	218	28.0 (24.8–31.1)	779
*G. vaginalis*	301	38.6 (35.2–42.1)	779
*M. hominis*	78	10.0 (7.9–12.1)	779
*L. iners*	489	62.8 (59.4–66.2)	779
Any lactobacillus	741	95.1 (93.6–96.6)	779
BV-score 1	19	3.7 (2.0–5.3)	518^4^
BV-score 2	49	9.5 (6.9–12.0)	518^4^
BV-score 3	7	1.4 (0.4–2.3)	518^4^
BV-score 4	44	8.5 (6.1–10.9)	518^4^
BV-score 5	26	5.0 (3.1–6.9)	518^4^
BV-score ≥ 2^3^	126	24.3 (20.6–28.0)	518^4^
*Candida*			
*C. albicans*	222	28.5 (25.3–31.7)	779
*C. glabrata*	55	7.1 (5.3–8.9)	779
*C. krusei*	36	4.6 (3.1–6.1)	779
*Candida* species	301	38.6 (35.2–42.1)	779
Other *Mycoplasma* and *Ureaplasma*			
*M. spermatophilum*	1	0.1 (0.01–0.8)	779
*U. urealyticum*	152	19.5 (16.7–22.3)	779
*U. parvum*	427	54.8 (51.3–58.3)	779

This table shows all detected organisms. No cases of *T. pallidum* or *M. pneumoniae* were found.

1 High-risk HPV types: HPV 16, 18, 31, 33, 35, 39, 45, 51, 52, 56, 58, 59, 66 and 68a/b

2 STI-positives include women positive for *M. genitalium*, *C. trachomatis*, *N. gonorrhoeae*, *T. vaginalis*, *T. pallidum* or HSV-1/2

3 BV-score takes low levels of lactobacillus and positivity for *G. vaginalis*, *A. vaginae* and *M. hominis* into account

4 BV-scores could only be calculated for samples with quantifiable *Lactobacillus*

**Table 3.** Correlations between HPV and STI.

	HPV-positive (*n* = 257)			High-risk HPV-positive (*n* = 172)		
Organism	*n*	% (95% CI)	*p*-value	*n*	% (95% CI)	*p*-value
STI						
*** C. trachomatis***	**5**	**1.9 (0.3–3.6)**	**0.004**	**4**	**2.3 (0.8–6.2)**	**0.010**
*T. vaginalis*	6	2.3 (0.5–4.2)	1.000	5	2.9 (0.4–5.4)	0.567
*N. gonorrhoeae*	3	1.2 (0.3–3.7)	0.338	1	0.6 (0.03–3.7)	1.000
*** M. genitalium***	**6**	**2.3 (0.5–4.2)**	**0.018**	2	1.2 (0.2–4.6)	0.692
** HSV-2**	**10**	**3.9 (1.5–6.3)**	**<0.001**	**8**	**4.7 (1.5–7.8)**	**0.001**
HSV-1	1	0.4 (0.02–12.5)	1.000	1	0.6 (0.03–3.7)	0.527
**STI-positive^1^**	**28**	**10.9 (7.1–14.7)**	**<0.001**	**18**	**10.5 (5.9–15.0)**	**0.011**
BV						
*A. vaginae*	**85**	**33.1 (27.3–38.8)**	**0.028**	**60**	**34.9 (27.8–42.0)**	**0.027**
*G. vaginalis*	**114**	**44.4 (38.3–50.4)**	**0.023**	**83**	**48.3 (40.8–55.7)**	**0.004**
*M. hominis*	**48**	**18.7 (13.9–23.4)**	**<0.001**	**39**	**22.7 (16.4–28.9)**	**<0.001**
Any lactobacillus	242	94.2 (91.3–97.0)	0.381	160	93.0 (89.2–96.8)	0.161
BV-score ≥ 2^2,3^	45	27.3 (20.5–34.1)	0.323	33	30.6 (21.9–39.2)	0.101
*Candida* species^4^	110	42.8 (36.8–48.9)	0.101	77	44.8 (37.3–52.2)	0.063
Other *Mycoplasma* and *Ureaplasma*						
* M. spermatophilum*	1	0.4 (0.02–12.5)	0.330	1	0.6 (0.03–3.7)	0.221
* U. urealyticum*	60	23.3 (18.2–28.5)	0.680	40	23.3 (16.9–29.6)	0.191
***U. parvum***	**172**	**66.9 (61.2–72.7)**	**<0.001**	**116**	**67.4 (60.4–74.4)**	**<0.001**

This table shows the correlations between STIs and HPV and high-risk HPV positivity. All *p*-values were calculated by Fisher’s exact test and are in reference to HPV-negative women.

1 STI-positives include women positive for *M. genitalium*, *C. trachomatis*, *N. gonorrhoeae*, *T. vaginalis*, *T. pallidum* or HSV-1/2

2 BV-score takes low level of lactobacillus and positivity for *G. vaginalis*, *A. vaginae* and *M. hominis* into account

3 BV-scores could only be calculated for samples with quantifiable *Lactobacillus*

4 None of the *Candida* subtypes showed a significant correlation with HPV and are therefore not listed separately

## Supplementary information

**Supplementary Table 1.** Inclusion and exclusion criteria.

Inclusion criteria	Exclusion criteria
• Women visiting the antenatal care/family planning units of the study centers	• Pregnancy-related complications: Placenta praevia Severe preeclampsia Vaginal bleeding Contractions Premature rupture of membranes
• Age 18–45 years	
• Able to understand the study	
• Consent given	
• Completion of the study procedures	• Multiple abortions in the past
